# Selective Vulnerability to Neurodegenerative Disease: Insights from Cell Type-Specific Translatome Studies

**DOI:** 10.3390/biology13020067

**Published:** 2024-01-23

**Authors:** Walker S. Jackson, Susanne Bauer, Lech Kaczmarczyk, Srivathsa S. Magadi

**Affiliations:** 1 Wallenberg Center for Molecular Medicine, Linköping University, 581 85 Linköping, Sweden; susanne.bauer@liu.se (S.B.); lech.kaczmarczyk@liu.se (L.K.); srivathsa.magadi@liu.se (S.S.M.); 2 Department of Biomedical and Clinical Sciences, Linköping University, 581 85 Linköping, Sweden

**Keywords:** Huntington’s disease, Alzheimer’s disease, prion disease, amyotrophic lateral sclerosis, RiboTag, bacTRAP, scRNAseq, snRNAseq

## Abstract

**Simple Summary:**

Neurodegenerative diseases, such as Alzheimer’s, Parkinson’s, and Huntington’s diseases, cause immense suffering to patients and their families. Despite an urgent need for therapies, they are still lacking, partly due to an incomplete understanding of the mechanisms involved. For example, it is unknown why specific brain regions are affected while others are not, a feature known as selective vulnerability. A better understanding of how certain regions resist disease, while others fail, could lead to new therapies. This review discusses ten studies that analyze gene expression changes in specific brain cell types as they respond to the early stages of four types of neurodegenerative disease. It concludes with a summary of recurring themes that address questions about the mechanisms behind selective vulnerability.

**Abstract:**

Neurodegenerative diseases (NDs) manifest a wide variety of clinical symptoms depending on the affected brain regions. Gaining insights into why certain regions are resistant while others are susceptible is vital for advancing therapeutic strategies. While gene expression changes offer clues about disease responses across brain regions, the mixture of cell types therein obscures experimental results. In recent years, methods that analyze the transcriptomes of individual cells (e.g., single-cell RNA sequencing or scRNAseq) have been widely used and have provided invaluable insights into specific cell types. Concurrently, transgene-based techniques that dissect cell type-specific translatomes (CSTs) in model systems, like RiboTag and bacTRAP, offer unique advantages but have received less attention. This review juxtaposes the merits and drawbacks of both methodologies, focusing on the use of CSTs in understanding conditions like amyotrophic lateral sclerosis (ALS), Huntington’s disease (HD), Alzheimer’s disease (AD), and specific prion diseases like fatal familial insomnia (FFI), genetic Creutzfeldt–Jakob disease (gCJD), and acquired prion disease. We conclude by discussing the emerging trends observed across multiple diseases and emerging methods.

## 1. Introduction

Imagine the fright of finding yourself in an unknown room, surrounded by unfamiliar people with unknown intentions, with no memory of how you got there. Imagine the frustration of not being able to control your body movements enough to get dressed. Imagine feeling so tired that you hallucinate, but nonetheless cannot obtain rejuvenating sleep. People with NDs face these challenges as their health gradually declines until their premature death. NDs not only have societal and economic consequences but also bring intense personal suffering. Sadly, the search for treatments is hindered by our limited knowledge of the diseases’ underlying molecular mechanisms.

NDs are widely thought to be caused by the misfolding of specific proteins that eventually clump together into aggregates. Although the disease-causing proteins are widely expressed, they tend to damage specific regions, a phenomenon known as selective vulnerability. Some of the proposed mechanisms explaining selective vulnerability include how the misfolded proteins or aggregates spread through the brain, how brain regions are connected in neural networks, and how protective or detoxifying proteins are regionally distributed [1,2,3,4,5,6]. A better understanding of these and other mechanisms could open new avenues for therapeutic discoveries. For over two decades, researchers have investigated changes to gene expression as an indicator of how vulnerable and resistant regions respond to disease. Given the diverse cell types in each region, analyzing them individually can enhance our understanding selective vulnerability. Among many tools for cell-specific studies in brain tissues, this article emphasizes CSTs in mouse models, which have given valuable ND insights, yet lack comprehensive reviews. While CST studies of single cell types are informative [7,8,9], this review is focused on studies including multiple cell types, especially neurons. In some instances, the discussion is supplemented with results from scRNAseq-based experiments.

In this review, our objective is to contrast vulnerable cells with cells that are not vulnerable, which we call resistant. In the clinical neurology setting of the ND field the term “resistant” is often used in the context of the seemingly similar term “resilient”. Definitions of these terms and examples of their proper use have been proposed to limit confusion [10]. In short, resistance refers to brains that do not contain pathology (often defined as protein aggregates) despite being old enough to, while resilience refers to brains that function normally despite containing pathology [10]. This convention is quite useful when considering NDs at the whole-brain level [11,12,13,14,15,16]. However, at the cellular level, the presence of aggregates within a cell does not always mean it is unhealthy or not functional, and the absence of aggregates does not mean a cell is healthy. Moreover, some NDs produce extracellular aggregates. Should a healthy cell near an extracellular aggregate be considered resistant or resilient? Furthermore, a cell may change its expression of certain genes in response to the erosion of the neuronal network it functions in, but still be a healthy cell. Is that cell resistant or resilient…or both? These are important questions and results from the studies described in this review could be useful to improve our understanding of them. However, debating these distinctions would greatly expand the length of this review. To streamline this review, we classify cells as vulnerable if they die or exhibit phenotypic changes, such as a reduction or loss of expression of genes that mark that cell type, and classify all other cells as resistant.

The first section contrasts CST methods with the more widely used scRNAseq and single nuclei RNAseq (snRNAseq) methods. Section two examines two ALS-focused CST studies: one juxtaposing neurons and glia, the other comparing two similar neuron types with differing vulnerability. The third section delves into studies on three prion diseases, exploring how a single protein affects different brain areas based on the misfolding trigger. In the fourth section on HD, one study utilizing bacTRAP in a mouse model and snRNAseq in both mouse and human is compared to another standalone snRNAseq study. A third HD study used RiboTag to analyze a brain region typically considered to be resistant. The fifth section covers AD: one study used bacTRAP in a novel way and its results are compared to human snRNAseq studies. Table 1 contains the experimental details of the key studies in this review. The concluding section identifies recurring themes, offering some potential answers to the questions raised in Box 1. 

Cells undergoing stress, particularly in the context of disease, often change their expression of certain genes, a feature we call a response. A very simple way to compare how different cell types respond to ND is to compare the number of differentially expressed genes (DEGs). However, comparisons of the number of DEGs should only be made within a study, since there are important differences between studies, such as disease stage, statistical thresholds, and statistical power. It is also important to recognize that a small change in a highly expressed gene may still be impactful. Furthermore, the basal expression levels of genes may affect their vulnerability without changing in response to disease, for example, protective factors that are naturally high in a certain cell type. Nonetheless, it remains uncertain whether vulnerable cells exhibit more DEGs than their resistant counterparts, thereby serving as a marker for them, a question that this article seeks to address.

Finally, there is an inherent assumption of disease mechanism conservation between human and mouse models, although the conservation is not complete. One must be mindful that human samples are typically collected at the terminal stage of disease (or post-mortem), after severe degeneration has occurred, and with varying time intervals between death and the removal of brain tissue. In contrast, mouse studies often probe earlier disease stages with minimal degeneration and no postmortem delay. Therefore, if changes evident in mouse studies are not found in human tissues, it is not just a potential variation between species; another plausible reason could be that these changes are masked in studies of advanced-stage human samples.

Box 1 **Questions addressed in the gene expression studies.**

1. How does a specific cell type respond to different diseases?

2. Is there a consistent gene expression response in vulnerable cells?

3. Do resistant cells exhibit any response? If so, is this response conserved?

4. Do pathways consistently exhibit the same response across cells (e.g., always increase)?

5. Does a particular misfolded protein elicit uniform responses across various cell types?

6. Is there a consistent cell-type response to various forms (e.g., mutants) of a single misfolded protein?

7. Given NDs result from misfolded proteins, is the unfolded protein response (UPR) more pronounced in vulnerable or resistant cells during early stages?

8. Can the number of DEGs or type of DEGs (e.g., based on function, pathway, etc.) indicate vulnerability?

9. Do gene expression changes indicate gene expression dysfunction?

10. Do vulnerable cells or regions exhibit higher levels of the disease-causing protein compared to resistant ones?

## 2. Methods for Cell Type-Specific Gene Expression Analyses

The primary CST methods are RiboTag [17] and bacTRAP [18,19]. RiboTag uses Cre recombinase to direct the expression in cell types of interest, where an HA-tagged variant of Rpl22 (large subunit ribosome protein 22) is expressed from its native location in the genome. In contrast, bacTRAP uses a bacterial artificial chromosome (BAC) vector to express a GFP-tagged Rpl10a (large subunit ribosome protein 10a) from a random location in the genome. We have previously discussed the nuances between the two methods [20], but here we consider them practically equivalent and collectively categorize them as CST methods. 

**Figure 1:**
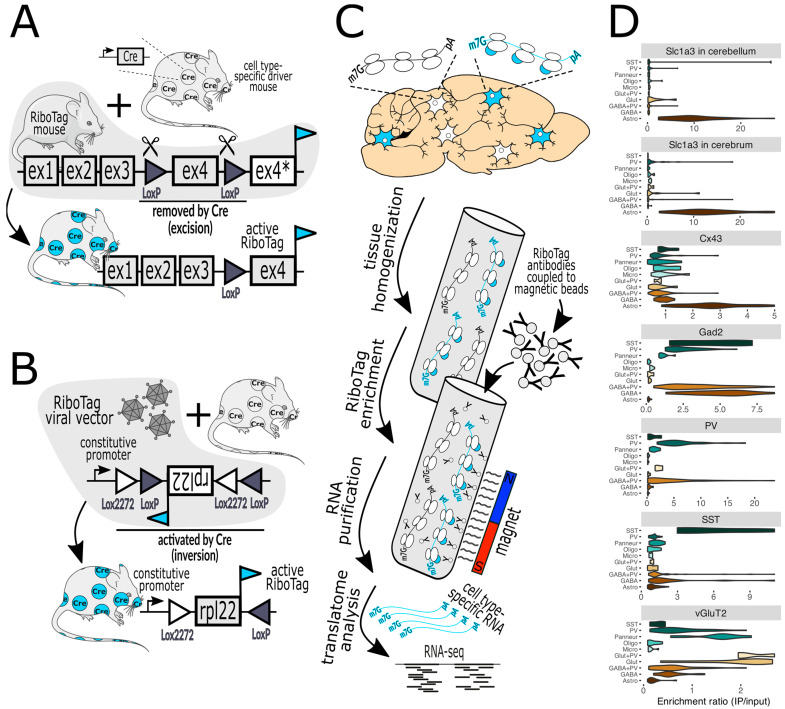
(**A**) In RiboTag mice, the native *Rpl22* gene has been engineered such that activation with Cre recombinase, expressed in specific cell types, results in the expression of Rpl22 fused with an HA epitope tag (cyan flag) specifically in those cells. Before Cre activation, the tagged exon 4 (ex4) is not expressed (ex4*). (**B**) A variant of this method employs a viral vector that delivers a RiboTag transgene that is also activated by Cre. The bacTRAP mice are practically the same as RiboTag mice regarding the capture of tagged ribosomes and are thus not shown. (**C**) illustrates the workflow, commencing with the homogenization of brain tissue, the capture of tagged ribosomes (marked with cyan-colored bubbles) with antibody-labeled paramagnetic beads, and then their subsequent analysis via RNA-seq (next-generation sequencing) and bioinformatics. (**D**) demonstrates a typical validation experiment wherein the purification of RiboTag-labeled ribosomes expressed in specific cell types (top label on each chart) results in the enrichment of marker genes of the desired cells and the depletion of markers of off-target cells (marker gene classes are on the left of each chart). For example, activation by Cre in cells expressing Slc1a3 (astrocyte marker) in the cerebellum or cerebrum (top two violin plots) facilitates the enrichment of astrocyte genes and the depletion of genes from neurons and other glial cells. Lower charts show the results when Cre is expressed by Cx43 (astrocytes), Gad2 (GABAergic neurons), PV (parvalbumin neurons), SST (somatostatin neurons), and vGluT2 (glutamatergic neurons). Marker gene classes include SST, PV, Panneur (pan-neuronal), Oligo (oligodendrocytes), Micro (microglia), Glut+PV (PV-expressing glutamatergic neurons), Glut (glutamatergic neurons), GABA+PV (PV-expressing GABAergic neurons), GABA (GABAergic neurons), and Astro (astrocytes). The Slc1a3 data were published in [29] and the rest were published in [30]. Mouse and neuron shapes were generously provided by https://smart.servier.com (accessed on 12 January 2024) under a CC 3.0 license.

Essentially, CST methods function by capturing epitope-tagged ribosomes using antibodies. Employing transgenic methods, ribosomes are tagged because the epitope-tagged ribosome protein integrates into ribosomes during assembly (Figure 1). Transgenesis lets researchers focus the expression of the tagged ribosomes in select cell types, where they function normally and translate mRNAs into proteins. The epitope tags facilitate the immune capture of the ribosomes and their associated mRNAs from brain homogenates (Figure 1). This design offers several advantages:

1. Tagged ribosomes are present only in target cells, providing an efficient separation of desired mRNAs from those of unwanted cell types.
2. The captured mRNAs reflect the cell’s translatome, and thus provide insights into the proteins being synthesized by the cell [21].
3. The method is compatible with frozen tissues, limiting batch variation between samples.

**Figure 2:**
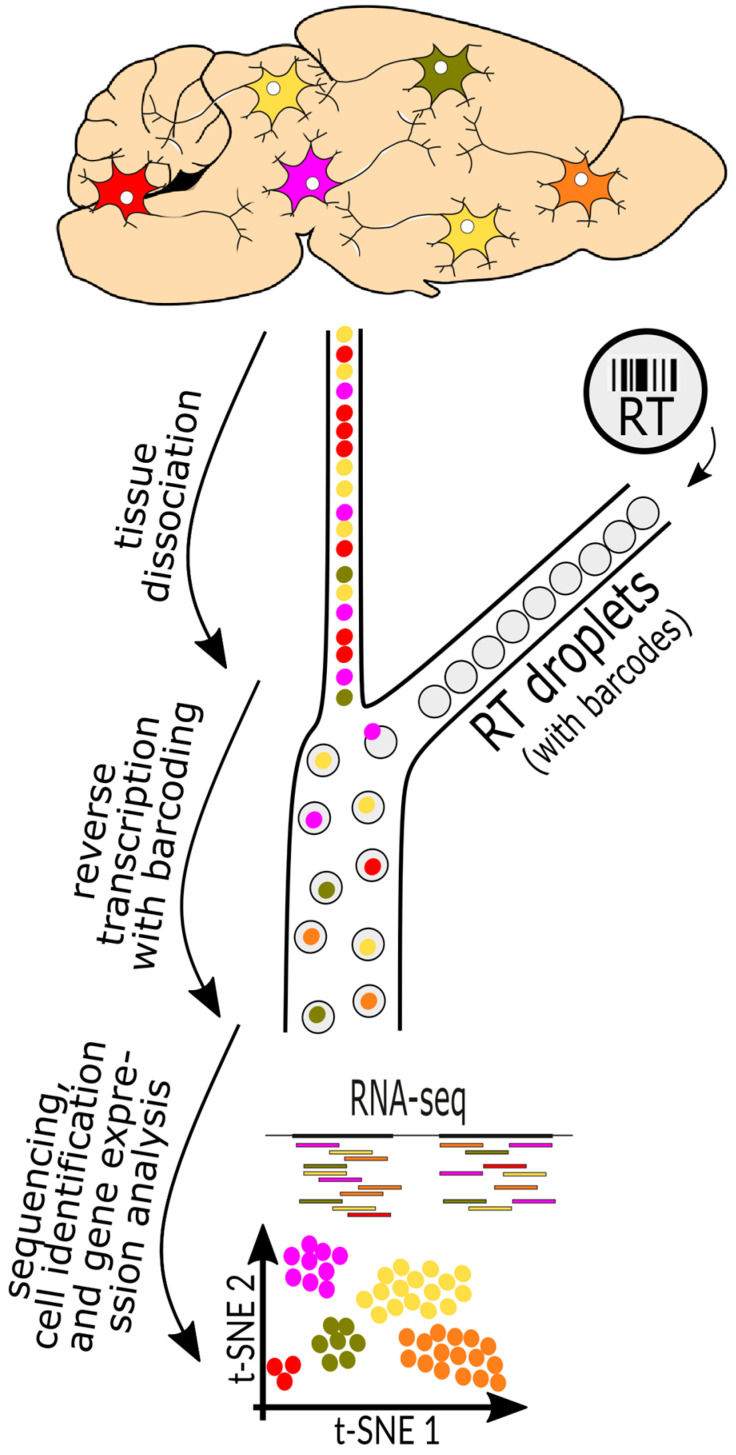
Workflow for scRNAseq and snRNAseq analyses. From the top down, cells of different types are marked in different colors. Cells or cell nuclei are gently dissociated and subjected to a device that orders them into a single file (left side, colored dots), and then merges each cell or nucleus with a single oil droplet (grey bubbles on the right) that carries barcodes that are unique to each droplet. The droplets are then pooled, amplified, and sequenced together, which diminishes batch effects. Features and similarities of each cell are then compared using t-SNE (t-distributed stochastic neighbor embedding) plots.

An alternative to CST methods is scRNAseq, where cells from a tissue are physically separated and the transcriptome of each cell is analyzed. Popular methods encapsulate cells in oil droplets, acting as micro-reaction chambers for converting mRNA to cDNA (Figure 2). The process includes the marking of each transcript (i.e., each molecule) to control for amplification artifacts, to assign each transcript to a specific cell (barcoding), and the conversion of each mRNA into cDNA (Figure 2). Due to their unique barcodes, the products from all cells are pooled before final amplification.

A key step in scRNAseq is isolating single cells, which is especially challenging for neurons in the brain, where fine processes such as dendrites and synapses are easily lost. Several dissociation methods exist that use physical or enzymatic dissociation mechanisms, but they can bias the sample quality and obscure mRNA level measurements. snRNAseq serves as a good alternative to scRNAseq, since nuclei are less prone to enzymatic and physical isolation artifacts than whole cells. Furthermore, snRNAseq has the advantage of being suitable for fixed and frozen samples, enabling studies of archived postmortem human brain samples, which are not possible with scRNAseq where fresh tissue is required. Several studies have shown significant similarity in transcriptomic analyses between scRNAseq and snRNAseq [22,23,24,25], although microglial activation genes were depleted in snRNAseq compared to scRNAseq studies in AD [26]. Furthermore, a comparison of different tissue dissociation protocols found differences in cell composition and further demonstrated the potential for biases in scRNAseq and snRNAseq [27]. For example, twice the number of genes per cell were detected with snRNAseq compared to scRNAseq [27]. A systematic analysis comparing different tissue dissociation protocols, cell lysis, sequencing, and data analysis led to the establishment of a toolbox that provides guidelines for customizing sc/snRNAseq protocols [28]. 

How do CST and sc/snRNAseq compare? Both methods face challenges with contamination from unintended cells [21]. CST analyzes a mix of related cell types, but captures approximately 15,000 genes per cell type, approaching the maximum number expected, since many genes are expressed only in certain organs or cell types. In contrast, sc/snRNAseq retrieves fewer genes but from distinct cells. A critical drawback of CST is the demand for artificial gene expression, usually delivered via transgenesis, rendering it unsuitable for human postmortem brain samples. A summary of the advantages and disadvantages of these methods is included in Table 2.

## 3. CST Studies of ALS 

ALS primarily targets subsets of motor neurons (MNs) in the motor cortex, brainstem, and spinal cord [31]. While most ALS cases are sporadic, there are many cases driven by mutations in several genes, of which the first to be discovered was superoxide dismutase 1 (SOD1) [32]. Interestingly, different SOD1 mutations can have different effects on its enzymatic function and aggregation propensity, leading to mutation-specific disease changes [33]. This, and the fact that historically SOD1 was the first gene associated with ALS, led to the production of several thoroughly studied SOD1 mutant mouse lines [34,35].

The first application of CST in ND was with bacTRAP [36] in the loxSOD1^G37R^ random integration mouse model of ALS [37], focusing on the MNs, astrocytes, and oligodendrocytes of spinal cords. Most of the study focused on the disease onset stage, marked at 8 months of a disease progression that reaches its terminal stage at 13.5 months. Transgene expression levels were measured and compared to the endogenous mouse SOD1 gene; mutant SOD1 was overexpressed 17-, 8-, and 21-fold in MNs, astrocytes, and oligodendrocytes, respectively. The authors then measured gene expression changes and found that MNs, the cells known to be most affected, had the highest number of DEGs at 260 (85% were increased), versus 108 for astrocytes, and 23 for oligodendrocytes. Of the MN DEGs that were increased, 10% had pathway features related to synaptic structures and cell junctions [26]. Genes related to the unfolded protein response (UPR) were also upregulated, along with the diminished expression of genes associated with ribosome biogenesis. This shift in gene expression might represent the cells’ adaptive strategy to reduce protein synthesis, potentially serving as a defense mechanism to counteract the detrimental effects of SOD1^G37R^ misfolding. Notably, the study did not find alterations in mitochondrial genes, in contrast to other studies discussed later in this review, where changes to ribosome and mitochondria biogenesis appear to be coupled. Nonetheless, it is noteworthy that there were 10 times more DEGs in MNs than in oligodendrocytes at this disease onset stage. According to the authors, the disparity in the number of DEGs, with MNs showing a high count and oligodendrocytes a considerably lower one, suggests that the disease begins in MNs and subsequently impacts oligodendrocytes as it progresses [26].

A more recent study of ALS employed bacTRAP to reveal differences between vulnerable and resistant MNs in layer 5b of the motor cortex [28] using SOD1^G93A^ transgenic mice [29]. New bacTRAP lines were created with promoters that were specifically active in layer 5b, based on anatomical data. Interestingly, two of the new mouse lines, built into BAC transgenes of *Colgalt2* and *Gprin3*, both expressed the bacTRAP protein in layer 5b of the motor cortex (M1). The bacTRAP-expressing neuronal populations had similar morphology and size, and both projected to the pons region of the brainstem, but Gprin3^+^ neurons also projected to the spinal cord. Importantly, with similar transgene expression levels in both cell types, by the time SOD1^G93A^ transgenic mice reached 110 days of age, approximately 40% of the Gprin3^+^ neurons died. Meanwhile, there was no noticeable decline in the number of Colgalt2^+^ neurons. This striking difference could be attributed to their intrinsic gene expression patterns. Gprin3^+^ neurons, even at baseline, exhibited an elevated propensity for oxidative phosphorylation but were notably lacking in genes that safeguarded against oxidative damage, and the SOD1^G93A^ transgene may have exaggerated this imbalance [28]. 

When expressing the SOD1^G93A^ transgene, Gprin3^+^ neurons underwent dramatic gene expression changes, particularly increasing their expression of an array of mitochondrial proteins, potentially amplifying oxidative damage through the increased production of hazardous oxidation catabolites [28]. It is hard to envision how this potentially dangerous response could be beneficial to these vulnerable neurons, but it is also improbable that this coordinated response is random. Also increased were genes encoding ribosomal proteins, and a similar coordination between ribosome and mitochondria biogenesis has been reported in HD [30]. Due to the high energetic expense required to generate ribosomes, it is logical for the biogenesis of ribosomes and mitochondria to be coordinated [31]. In this context, it could be that the primary response was to increase ribosome biogenesis, leading to the increased need for mitochondrial biogenesis and metabolic activity that doomed these neurons. Interestingly, the Colgalt2^+^ MNs also increased the expression of both mitochondrial and ribosomal proteins, but the increase was smaller in scale (about 30% less) and their higher baseline of protection from oxidative damage likely enabled them to fare much better than Gprin3^+^ neurons. Notably, both neuron types decreased their expression of genes related to synapse and axon morphogenesis, with a larger effect in the Gprin3^+^ neurons. The authors concluded that intrinsic properties of the Colgalt2^+^ MNs (i.e., the higher baseline expression of antioxidant genes) determined their resistance.

To summarize the results from the ALS section, the first study [36] indicated that the biogenesis of ribosomes decreased in the most susceptible cells, despite no concurrent decrease in mitochondrial biogenesis. This reduction was accompanied by a pronounced activation of the unfolded protein response (UPR). The second study [38] compared two neuron types that are strikingly similar. While the more susceptible neurons exhibited tenfold more differentially expressed genes (DEGs) than their resistant counterparts, the overall genetic alterations were similar. These changes in gene expression seemed to align with specific molecular pathways, most notably increased ribosome and mitochondrial biogenesis. It is important to note that when this analysis took place, 40% of Gprin3^+^ MNs had already succumbed. Hence, the observed changes in gene expression might either represent the adaptive strategies of the surviving neurons or be attributed to a change in the compared populations. Nonetheless, a consistent theme from both studies is that even the more resistant cells can exhibit as much disease-causing protein expression as the vulnerable ones, and the highest DEG counts typically arise in the latter. Interestingly, while the UPR was prominent in the first study, it was absent in the second, even when numerous neurons in the latter were evidently under stress.

## 4. CST Studies of Prion Diseases 

Prion diseases are another class of rare neurodegenerative diseases. While they are most infamous for cases caused by infection (e.g., “mad cow” disease) from misfolded forms of the prion protein (PrP), they can also be caused by the inheritance of mutations in the gene encoding PrP (*Prnp*) or from spontaneous PrP misfolding [4]. Although rare in humans, acquired prion diseases (i.e., those caused by infection) are a problem in farmed sheep and goats in Europe and in wild cervids in North America [39,40], a problem that is emerging in Northern Europe with new properties [41,42]. The diseases’ infectious nature was exploited to develop some of the first mouse models of neurodegenerative diseases. Work on rodent models made biochemical purifications of the infectious agent possible and led to the discovery of PrP, and in turn, *PRNP* [43,44]. This led to the discovery that multiple inherited neurodegenerative diseases including fatal familial insomnia (FFI), genetic Creutzfeldt–Jakob disease (gCJD), and Gerstmann–Straussler–Scheinker syndrome (GSS), are caused by certain mutations in *PRNP* [45,46,47]. Prion diseases cause damage in specific brain regions depending on the disease subtype [4,48,49]. Furthermore, the shapes of PrP aggregates and the abundance and size of spongiform degeneration “holes”, a hallmark of prion diseases, also vary according to the disease subtype. Interestingly, PV^+^ neurons, a subset of GABAergic inhibitory neurons, were reported to be highly vulnerable to prion diseases in humans and rodents [50,51,52], though less severely affected in FFI [48]. To uncover how different neurons respond to different misfolded conformers of the same protein, we recently reported studies that we have performed using RiboTag for the pre-onset disease stages of an acquired prion disease [30] and two genetic prion diseases [53]. Another study of acquired prion disease used a Cre-dependent TRAP method but detected changes only at the terminal stage [54]. Similarly, a scRNAseq study that focused on a near-terminal disease stage has been reported [55]. Although these reports provided novel insights into disease mechanisms, the results were focused on terminal disease stages, unlike all other studies described in this review, and thus will not be considered further.

In the first RiboTag study, we analyzed the translatome profiles of astrocytes and four neuron subtypes during a very early disease stage of acquired prion disease, 10 weeks of a 23-week disease course, long before neuropathological and electroencephalography changes were detected [30]. The disease model employed mice expressing wild-type PrP from the endogenous *Prnp* gene and the disease process was triggered by an injection of infectious prions. Surprisingly, PV^+^ neurons showed essentially no response (three DEGs). Neurons expressing somatostatin (SST), another subset of GABAergic neurons mostly non-overlapping with PV^+^ neurons, also showed essentially no response (one DEG). Interestingly, the broader category of GABAergic neurons revealed a more pronounced response, identifying 83 DEGs primarily associated with the circadian rhythm network and mitochondrial proteins. Glutamatergic excitatory neurons had a tempered response, with 38 DEGs primarily related to the cytoskeletal network, with a particular emphasis on spectrins. Of all the cells studied, astrocytes had the most marked response, revealing 139 DEGs. The majority of these were associated with the downregulation of ribosome and mitochondrial proteins. Notably, during these early stages of the disease, the UPR was not activated in any cell types, but it was evident when the disease reached clinical onset. Given that astrocytes displayed the highest number of DEGs, a question arises: were the astrocytes the most impacted, or were they simply adjusting to a changing brain? A more recent bacTRAP study of non-diseased mice offers some perspective [56]. This study revealed that astrocytes adjust to neuronal activity by amplifying ribosome production, which in turn augments translation near neuronal synapses. The ribosome changes were also accompanied by increases in mitochondrial proteins, and both increases and decreases in cytoskeletal proteins. Piecing these findings together, it is conceivable that reduced neuronal activity in certain GABAergic and/or glutamatergic neurons caused by prion disease would diminish the demands of translation in astrocytes, triggering a reduced expression of ribosomal and mitochondrial proteins. In such a scenario, the high number of DEGs in the astrocytes would not necessarily reflect a cell type being negatively affected, but rather signify an adaptive response to surrounding changes.

In the second study, we examined mouse models of FFI and gCJD, also at a pre-onset stage (9 months old) [53]. The mice were engineered to express mutations linked to FFI or gCJD by manipulating the mouse’s endogenous gene, resulting in knock-in (KI) mice [57,58]. These models were previously reported to primarily target the thalamus (FFI, [57]) and hippocampus (gCJD, [58]), regions that are rich in glutamatergic neurons. Both models also targeted the cerebellum, albeit less severely. Like the acquired prion disease study above, the inherited prion disease study used RiboTag with the same neuronal Cre lines, but separately examined the cerebellum and the remaining cerebrum. The data were revealing: like in the first study, PV^+^ neurons essentially did not respond, with only two or three DEGs in gCJD and FFI, respectively. Yet, a surprise came from the SST^+^ neurons, which exhibited a robust response, with 153 DEGs for gCJD and an even more significant 684 DEGs for FFI. A network analysis indicated that coordinated changes were induced by mTOR signaling, resulting in the increased synthesis of ribosomal and cytoskeletal proteins. Notably, this mTOR activity was absent in the previously discussed acquired prion study. Like in the acquired prion study, UPR was not triggered in either the FFI or gCJD models. However, since only the pre-onset stage was analyzed, the possibility of UPR manifesting at a later disease stage cannot be excluded. Compared to the acquired prion study, glutamatergic neurons in the genetic prion study had an even milder response (in the cerebrum, three DEGS in gCJD and 11 in FFI; in the cerebellum, two DEGS in gCJD and 19 in FFI). This was particularly interesting since GABAergic neurons express about half as much PrP as glutamatergic neurons, yet in both studies GABAergic neurons registered more DEGs. This result indicates that expression levels of a disease-causing protein do not strictly determine a neuron’s vulnerability.

A summary of the prion disease studies leads to several important conclusions. Since in both studies PrP was expressed from the native gene in its endogenous locus, PrP expression pattern differences cannot account for the different results reported in the two studies. Therefore, the first conclusion is that the same protein can unleash different gene expression responses within a specific cell type, depending on how the protein misfolds. Second, different cell types respond to the same misfolded protein form differently. Third, since glutamatergic neurons expressed PrP more highly than all other cell types studied, their mild response during prion disease suggests the most affected cells do not necessarily express the protein the highest. Fourth, vulnerable cell types (i.e., PV^+^ neurons) can have a very mild response with surprisingly few DEGs. Fifth, the UPR is not always induced during the early stages of ND.

## 5. CST Studies of HD

HD is caused by the expansion of a CAG repeat in exon 1 of the huntingtin (*HTT*) gene, which is translated into a polyglutamine tract. While repeat lengths of 6 to 35 CAGs do not cause disease, repeats of 36 to 39 CAGs are linked to HD with incomplete penetrance, and repeats with 40 or more CAGs cause HD with high penetrance at an average age of 40 years [59,60,61]. Early HD neuropathology is characterized by the selective degeneration of GABAergic medium spiny neurons (SPNs) of the striatum, which constitute around 95% of all striatal neurons, while other striatal cell types are usually spared [62,63]. Striatal SPNs can be further subdivided into two main subtypes based on their expression of dopamine receptors (Drds). Drd2-expressing SPNs of the indirect pathway (iSPN) show higher vulnerability than Drd1-expressing SPNs of the direct pathway (dSPN) [64]. This interplay of the regional and cell type-specific vulnerability of neurons to mutant Htt (mHtt) is a subject of great interest in the HD field, but our understanding of the underlying mechanisms remains incomplete. Fortunately, recent studies employing bacTRAP, RiboTag, and snRNAseq methods have provided new details of cell type-specific responses to mHtt, as elaborated in the following section.

Since HD is strictly genetic, it is reasonable to assume that there is a common molecular mechanism in all patients, and knowledge derived from studies of genetically engineered mice may be relevant for HD patients. The first genetic mouse model with a disease-relevant phenotype, the R6/2 model, was generated by injecting a fragment of the mutant *HTT* gene from a human with HD into the pronucleus of a fertilized mouse oocyte, where it integrated into a random location [65]. These mice develop a severe neurodegenerative disease that leads to death in young adult mice, at approximately 20% of their normal lifespan. In pursuit of a more accurate model, researchers employed another strategy: a long CAG repeat was inserted into the mouse *Htt* gene in its native location in the genome, resulting in KI mice. This has been performed by multiple labs with one of the key differences between models being that some included various amounts of human DNA sequences [66,67,68], whereas others used only the mouse *Htt* sequence [69,70]. Although these seemingly subtle differences can impact disease severity, the strongest determinant of severity is the length of the CAG repeat, where 150 to 200 triplets result in mild neurological disease, even at an advanced age, with little neuronal loss and only subtle markers of neurodegeneration. Despite these apparent shortcomings, the KI models cause gene expression changes detectable in crude tissue lysates reminiscent of those detected in similar preparations of human HD samples [71], offering valuable insights into the disease’s molecular mechanisms.

### 5.1. Vulnerable and Resistant SPNs Have Surprisingly Similar Responses to mHtt

Here we focused predominantly on the results from three recent studies using CST and snRNAseq to examine gene expression changes in KI mouse models of HD. The first study, by Lee et. al., employed the bacTRAP method to scrutinize changes in cell type-specific translatomes within the striatum [72]. This was conducted in an allelic series of HD KI mice, with a gradient of CAG lengths between 20 and 170 repeats [68], as well as in the similar zQ175DN model [66,67]. This study further used snRNAseq to analyze the features of striatal nuclei from zQ175DN mice as well as from humans with different degrees of HD severity, ranging from over 50% to nearly 100% SPN loss. We then discuss results reported by Malaiya et al. [73], who performed snRNAseq on striata from zQ175DN mice. Last, we compare these findings to a recent study conducted in our lab [74], wherein RiboTag was used to analyze cell type-specific expression changes in the cerebrum and cerebellum of pre-symptomatic HdhQ200 KI mice.

The cell type-specific analysis of gene expression changes using both bacTRAP and snRNAseq data revealed a high overlap in DEGs between dSPNs and iSPNs, which was conserved across HD models and prominent in human samples [72,73]. bacTRAP-derived translatome data revealed that while iSPNs showed consistently more DEGs, as may be predicted for more vulnerable neurons, more than 50% of dSPN DEGs were also differentially expressed in iSPNs [72]. When comparing bacTRAP and bulk RNA data from KI mice striata [71], iSPNs had the highest similarity. This suggests that, in the early stages, iSPNs contribute significantly to the overall gene expression changes witnessed in the total striatum [72]. Corroborating these insights, Malaiya et al.’s snRNAseq study revealed minimal pronounced differences between the SPN subtypes [73]. Shared DEGs between both SPN subtypes exhibited similar shifts in both direction and magnitude. Together, this suggests that the selective vulnerability of iSPNs versus dSPNs might not stem from differential gene expression changes [67]. Strengthening these findings, the congruence between human and mouse snRNAseq results and the bacTRAP data was evident [66,67]. This validates the credibility of the bacTRAP findings and indicates that a significant portion of gene expression changes originates at the transcription level.

Notably, the snRNAseq analysis of zQ175DN striatal tissue from both studies identified a third cluster of spiny neurons, which the authors proposed represents a recently characterized SPN subtype dubbed “eccentric” medium spiny neurons [75]. These SPNs are marked by their distinct expression of *Otof*, *Pcdh8*, *Cacng5*, *Casz1* [75], and *Col11a1* [76] and generally showed similar DEGs to dSPNs and iSPNs, including the downregulation of striatal markers and genes related to synaptic function [72,73], but they also showed opposing directionality in several DEGs [72]. These findings of a novel cell type highlight an important advantage of snRNAseq over CST methods. 

### 5.2. Functional Enrichment in Medium Spiny Neurons

Functional analysis of zQ175DN bacTRAP DEGs revealed both overlapping and SPN subtype-specific pathway changes [72]. Both SPN subtypes showed strong similarities in enriched KEGG pathways among their downregulated genes, including pathways related to dopaminergic synapse, long-term potentiation, circadian entrainment, and amphetamine addiction. dSPN-specific pathways included autophagy and circadian rhythm. In contrast, the pathway enrichment analysis for upregulated genes indicated a more cell type-specific response. dSPNs exhibited an enrichment of ND-related pathways, ER protein processing, and proteasome, whereas iSPN-specific upregulated genes were enriched for synapse-related pathways, choline metabolism, the regulation of the actin cytoskeleton, and Relaxin and Erb signaling pathways.

### 5.3. mtRNA Release and Innate Immune Response Activation as Potential Mechanisms Underlying the Selective Vulnerability of iSPNs

A remarkable discovery by Lee et al. was the elevated levels of mitochondrial-encoded RNA (mtRNAs) in both iSPNs and dSPNs, as shown by both bacTRAP and snRNAseq data sets [72]. Since mtRNA is typically confined to the mitochondria and not associated with ribosomes, the authors argue that the capture of mtRNAs by bacTRAP suggests that there is mitochondrial dysfunction that causes the release of mtRNA into the cytosol, where it attaches to cytosolic ribosomes. Supporting the idea that this is not merely a technical artifact, the amount of mtRNA captured was related to age and CAG repeat length in a manner that positively correlates with increased toxicity. Furthermore, cytosolic mtRNA was more pronounced in iSPNs, confirming a disease-specific phenomenon. This phenomenon was also paired with the reduced expression of genes linked to the oxidative phosphorylation pathway and the activation of the innate immune sensor protein kinase R (PKR) in iSPNs, alongside the elevated expression of interferon-responsive genes. With previous studies suggesting that mtRNAs can activate immune responses by binding to PKR [77,78], Lee et al. postulated a sequence in which mHtt causes cytosolic mtRNA buildup, leading to an immune response initiated by its direct interaction with PKR. 

In the same study, large amounts of extra-mitochondrial mtRNAs in the iSPNs and dSPNs of human HD samples were also detected using snRNAseq. While oxidative phosphorylation-related genes were also downregulated in this data set, this was observed in both SPN subtypes, unlike the iSPN-specific changes observed with bacTRAP data. The authors proposed that this may be due to regulation of oxidative phosphorylation genes at a translational level [79] which would not be detected in snRNAseq. Another plausible reason is that the mouse data were derived from young (barely affected) tissues while the human samples were much more severely affected, with a high proportion of neurons already dead. Interestingly, the bacTRAP data of dSPNs showed an enrichment of oxidative phosphorylation pathway genes among upregulated DEGs. The authors propose that this, together with a delayed increase in mtRNA in dSPN, may play a role in the selective vulnerability of iSPNs versus dSPNs [72]. However, Malaiya et al.’s snRNAseq study opted to exclude nuclei with mtRNA reads, concerned these might be technical artifacts [73]. Therefore, additional experiments to specifically detect mtRNA release in vivo are warranted.

### 5.4. Loss of Neuronal Identity in the Striatum 

An important finding across these HD striatal studies was the early downregulation of striatal marker genes, indicating a loss of cellular identity. A previous analysis of bulk striatal mRNA indicated that striatal markers, and in particular iSPN-specific genes, showed a strong negative association with CAG repeat length [71]. The cell type-specific translatome and transcriptome analyses by Lee et al. further revealed that the downregulation of striatal marker genes (*Scn4b*, *Arpp21*, *Gpr6*, *Pde10a*) occurred not only in SPNs, but in most striatal cell types including astroglia, oligodendrocytes, and cholinergic interneurons, which are generally resistant to HD [72]. The authors suggested this may be due to mHtt affecting common regulators in these cell types [72]. Similarly, Malaiya et al. found a pronounced downregulation of pan-striatal and SPN-specific marker genes (*Ppp1r1b*, *Pde10a*, *Rgs9*) using snRNAseq in all SPN subtypes [73]. The authors further identified the cell type-specific identity modules of co-expressed genes, using an adapted approach of weighted gene co-expression network analysis [73]. This revealed that, besides the downregulation of identity module genes in their respective cell types, cell identity genes were aberrantly upregulated in incorrect cell types. This included the upregulation of SPN identity genes in astrocytes and oligodendrocytes and vice versa. The number of identified iSPNs was further reduced by approximately 30% in 14–15-month-old mice, despite the absence of neuronal death at this disease stage, which the authors attributed to a loss of cell identity markers rather than a loss of neurons [73].

### 5.5. Predicted Regulators of Gene Expression Changes in HD

It was previously shown that HTT facilitates PRC2 activity [80], promoting cell fate specification. Consequently, a proposed mechanism of HD is that mHTT induces the de-repression of non-MSN genes and genes associated with neurodegenerative processes [81]. Supporting this idea, the CAG-dependent downregulation of several transcription and chromatin factors, including the PRC2 component *Ezh2*, was found as early as 2 months in RNAseq data of whole striata from KI mice [71]. Furthermore, a comparison of snRNAseq and PRC2 chromatin-histone immunoprecipitation data found an enrichment of PRC2 target genes in several cell type-specific modules [73]. These PRC2-regulated modules showed progressive, age-related dysregulation and ectopic expression patterns in HD mice, leading the authors to propose that the loss of function of PRC2 in HD is an underlying mechanism affecting cell identity maintenance in all striatal cell types [73]. Based on bacTRAP-derived expression changes in KI mice, PRC2-related genes were among the top predicted regulators of downregulated genes in both SPN types and corticostriatal projection neurons [72]. Additional predicted top regulators for DEGs shared between SPN subtypes included several genes previously implicated in HD, including the transcriptional regulators *Crem*, *Creb1*, and histone demethylase *Kdm5b* for shared upregulated DEGs, and *Wt1* [82] and *Rarb* (retinoic acid receptor beta) [83] for shared downregulated DEGs. Predicted regulators with SPN subtype-specific roles included *Vdr* and *Foxo3* for dSPN-only upregulated genes, and *Wt1* for iSPN-only upregulated genes [72]. 

### 5.6. RiboTag Analysis of a Brain Region less Vulnerable to HD 

In the third analysis of HD, we applied RiboTag to study 9-month-old HdhQ200 knock-in model mice [69,70], but in this case with a focus on the cerebellum [74], which was performed in parallel with the FFI and gCJD study described earlier [53]. The cerebellum was of interest since Purkinje cells (GABAergic neurons) are vulnerable in HD, whereas granule cells (glutamatergic neurons) are resistant [84,85,86,87]. The remaining part of the brain, sans the olfactory bulb, was also studied and referred to as the cerebrum. GABAergic and glutamatergic neurons were studied in both regions, whereas PV^+^ neurons were also studied in the cerebrum samples [74]. 

First, the expression of *Htt* was measured across eight brain regions and it was determined that the striatum had very low expression levels, second only to the cerebellum, indicating that vulnerable regions are not susceptible due to their high expression of mHtt. Next, differential gene expression analyses of RiboTag-captured mRNAs revealed that, in the cerebrum, GABAergic neurons had the most DEGs, with 62. Interestingly, despite the cerebrum containing numerous brain regions, the gene expression changes had many similarities to the striatum-focused studies described above, including decreases in synaptic protein genes and striatal cell type markers such as *Penk*, *Drd1*, and *Drd2* [74]. The detection of these apparent striatum-originating signals was likely facilitated by the fact that the striatum had a very high density of RiboTag-expressing neurons (approximately 95%), whereas other regions had 20% or fewer neurons expressing RiboTag. Interestingly, in the cerebellum glutamatergic neurons had 626 DEGs compared to only 12 in GABAergic neurons. The low number of DEGs in glutamatergic cerebellar neurons from CJD (2) and FFI (19) indicates that the large number seen in HD was not simply due to the high homogeneity of cerebellar granule cells. Since granule cells are thought to be resistant, at least compared to Purkinje cells, the concept that the number of DEGs is indicative of a neuron type’s sensitivity is once again contradicted. However, some of the changes are not expected to be made by healthy neurons. For example, cyclin D1 (*Ccnd1*), a gene whose activation in neurons often precedes apoptosis, was one of the most differentially expressed DEGs. Furthermore, PRC1-related genes, overlapping but with some distinction to PRC2, were implicated as causing many of the granule cell DEGs, implying there is a similar responding mechanism in vulnerable striatal neurons and resistant cerebellar neurons. Importantly, this strong response in granule cells was not detected in the FFI and CJD cerebella described earlier, indicating that it was not a general response to neurodegeneration but specific to polyglutamine toxicity. Notably, in contrast to the prominent detection of mitochondrial-encoded RNAs in the bacTRAP study, the RiboTag study did not report such a finding in any cell type.

Taken together, the results from these studies of HD suggest that a loss of cell identity affects nearly all striatal neuron types, even at pre-symptomatic stages, and the loss of function of PRC2 underlies many of these gene expression changes. Interestingly, vulnerable and resistant SPN subtypes showed very similar gene expression changes across studies and methods. Finally, the number of DEGs may not always be indicative of a cell’s vulnerability. While in the striatum there were more DEGs in iSPNs than dSPNs, in the cerebellum there was a surprisingly high number of DEGs in the resistant granule neurons but very few DEGs in GABAergic neurons, which includes the vulnerable Purkinje neurons.

## 6. CST Studies of AD

AD diminishes cognition and memory by attacking brain regions linked to those functions, especially the cornu ammonis 1 (CA1) region of the hippocampus and layer II of the entorhinal cortex (ECII) [88,89]. The locus coreuelus is also affected very early and with a high impact [90,91,92,93], but is understudied, possibly since its connection to cognition and memory, the most studied clinical features of AD, is not well established. As with other NDs, AD neuropathology is marked by the aggregation of specific proteins. Amyloid beta (AB) peptides, typically consisting of 40 or 42 amino acids, are derived from proteolysis of the amyloid precursor protein and clump together into extracellular amyloid plaques when their stoichiometric balance is perturbed [94,95,96]. Similarly, the microtubule-associated protein tau forms aggregates that are toxic. Tau has six typical isoforms due to alternative splicing, with zero, one, or two inserts at the N-terminus, while its C-terminal half typically contains either three or four repeating units (3R and 4R, respectively) of microtubule-binding domains [2,97]. Furthermore, tau is subject to multiple post-translational modifications including acetylation, ubiquitylation, and phosphorylation [2]. The imbalance of 3R/4R ratios and the subsequent aggregation and modification results in intracellular tau aggregates known as neurofibrillary tangles (NFTs), which are widely thought to be toxic in AD and related tauopathies [2,98,99]. While amyloid plaques appear to precede NFTs, they tend to be widespread and not selective, whereas the location of NFTs more closely correlates with neuronal loss [2,89,100].

We conclude our review by considering three studies on AD [101,102,103], focusing most on a study where bacTRAP was used in a unique way for AD [101]. In short, rather than focusing on comparisons between diseased and control animals, the authors focused on defining the characteristics of vulnerable and resistant neurons as they aged. This was a clever strategy, since most cases of AD are not genetic and are thus difficult to model in mice. However, advanced age is the leading risk factor for non-genetic forms of AD and can be conveniently controlled in mouse studies. The bacTRAP data were subsequently integrated with human data to develop a computational model that revealed neuronal structural changes and a connection with alpha-synuclein (A-syn) [101]. The AD section ends with two snRNAseq analyses of patient brains that revealed changes to myelination [102] and identified a novel marker of vulnerable neurons [103]. 

To generate new knowledge about selective vulnerability in AD, bacTRAP mice were generated to selectively study two types of vulnerable neurons, namely excitatory neurons in CA1 and ECII, as well as five types of resistant neurons, including excitatory neurons in the CA2, CA3, and dentate gyrus of the hippocampus, and excitatory neurons in visual and somatosensory cortical areas [101]. Translatome signatures were created for each neuron type in non-diseased mice at 5, 12, and 24 months of age, with the rationale that aging can inform about AD mechanisms, since it is an age-dependent disease. These signatures were validated by comparing each, one by one, to the transcriptome signatures of 205 human brain regions. Remarkably, neuronal signatures for each of the seven mouse regions most closely matched the signatures of the corresponding human region. The authors then built new computational models to combine the mouse signature data with human genome-wide association study (GWAS) data based on NFT pathology that had been sculpted with algorithms to include cell type-specific functional network information. They then created a new test data set composed of bacTRAP-derived translatomes of ECII neurons in 6-month-old mice genetically engineered to generate AB plaques [104] (the authors studied only this cell type in the context of the disease). This analysis detected 1936 DEGs compared to non-transgenic controls. Contrary to many of the studies described above, the ribosomes were not changed, but rather several cytoskeletal and synaptic proteins were altered, similar to some of the previous studies described above. Beyond the cytoskeletal changes seen in many NDs, this study also yielded a potential explanation for a connection between A-syn aggregates and AD, as explained below.

Since the computational model included GWAS data tied to the NFT burden in AD patients, the authors were well-positioned to detect genes involved in tau processing. Indeed, they found that PTBP1 (polypyrimidine tract binding protein 1) regulates tau splicing and its dysregulation causes an imbalance in 3R/4R tau levels. Interestingly, PTBP1 also regulated A-syn. A-syn is most infamous for its prominent role in a range of NDs known as synucleinopathies, including Parkinson’s disease, Lewy body dementia, and multiple systems atrophy. However, it was also previously associated with AD [105,106], although the molecular mechanism was poorly understood. A study by Roussarie et al. implicates A-syn’s established role at the synapse, where the neuronal cytoskeleton is most dynamic, as the key to its connection to AD [101]. The authors concluded that high vulnerability may be anchored in ECII neurons because their processes are highly dynamic, which can easily become unbalanced with age or an AB plaque burden, leading to tau imbalance, toxic NFT formation, and its eventual spread to secondarily vulnerable regions [2,89,100].

Interestingly, the snRNAseq studies revealed different information about selective vulnerability in AD [102,103]. In one of the first such studies of the prefrontal cortex, it was concluded that early in disease there are many cell type-specific gene expression changes, especially downregulations, but the differences become diminished as the disease progresses [102]. Furthermore, pathways related to myelination, in both oligodendrocytes and neurons, were prominently affected [102]. In a later study using snRNAseq from human samples [103], it was determined that RORB (Retinoic acid receptor-related Orphan Receptor B), a protein best characterized for its role in cortical development [107,108,109], served as a highly useful marker to identify specifically those neurons in ECII that are most vulnerable [103]. It was determined that these RORB^+^ cells in ECII have very similar signatures as a cell type in the superior frontal gyrus that is also vulnerable. Histological experiments revealed that vulnerable RORB^+^ cell types had at least two broadly different morphologies in both regions, indicating that cell shape alone (and thus probably also firing properties) is not sufficient to distinguish vulnerable from resistant cells. Interestingly, in both snRNAseq studies, inhibitory neurons showed little, if any, vulnerability [102,103].

Thus, through these three studies of AD, we have learned that functional weaknesses include the neuronal cytoskeleton and myelination, and that RORB is an excellent marker of vulnerable neurons. While these impressive results do not mean the selective vulnerability problem of AD is solved, they do outline the direction for additional experimentation. Indeed, all of these experiments neglected the locus coeruleus, which has NFTs even before ECII, the degeneration of which has been demonstrated to trigger the degeneration of hippocampal and cortical areas [90,91,92,93]. Using bacTRAP or RiboTag in RORB^+^ neurons in multiple regions, especially ECII, CA1, and the locus coeruleus, may build on the foundational knowledge provided by the studies reviewed here. 

## 7. Conclusions

### Notable Trends

In our survey of CST studies, we noticed some interesting trends. For example, the expression level of a disease-causing protein is often high in resistant cells or regions and low in vulnerable cells or regions, indicating that selective vulnerability is not simply a result of how highly the misfolded protein is expressed (both ALS studies [26,28], both prion studies [30,53], and the Bauer et al. HD study [74]). Furthermore, we highlighted examples of how a single misfolded protein can impact specific cell types in unique ways (all the CST studies), and that different variants of a protein can affect a single cell type differently (the prion studies [30,53], see also [110]). An unexpected result was that the UPR is often not activated in the early stages of ND. This suggests that the UPR is activated only late in NDs and that therapies aimed at controlling the UPR may not help during early disease stages. Interestingly, cell identity genes are consistently changed in HD. This could be caused by the diminished maintenance of mature, fully differentiated neurons, giving the impression of cell cycle reentry, which is also reported in HD and other NDs. Although not unexpected, excitatory neurons appear more vulnerable to certain NDs (ALS and AD), while inhibitory neurons appear more vulnerable to others (HD and prion diseases).

Perhaps the most striking trend is that multiple ribosome proteins often change in the same direction. Sometimes they are increased, while in others they are decreased, and this happens in vulnerable and resistant cell types. What could be the purpose? One obvious reason is a change in mTOR signaling, but, in some cases, ribosome proteins changed without changes to mTOR. An alternative hypothesis is that cells make these changes to adjust their intracellular crowding, which would in turn modulate the mechanical properties of the cells [111,112]. This would be an interesting mechanism to complement the changes to structural proteins such as actin and spectrins, which were also observed multiple times. 

At the beginning of this review, we pondered the meaning of cell types having few or many DEGs. Through this survey, we saw that the most vulnerable neurons do not always have the most DEGs (e.g., PV neurons in prion diseases had very few DEGs), although they did in other contexts (e.g., ALS and HD). We also saw that when resistant cells respond to a disease, the DEGs or pathways often overlap with those in the vulnerable neurons. Furthermore, there was a wide range of the number of DEGs within and across studies. In the introduction, we listed several technical explanations for why the number of DEGs can vary across studies, but there could also be a biological reason. When a molecular pathway has lots of components, it may require many DEGs to thoroughly change its output. Likewise, a pathway with a few components may need a few DEGs to change its output, but such a pathway may still be important. Furthermore, if a certain pathway is engaged by different cell types, each cell type may manipulate different genes in that pathway with the same overall effect. Focusing on specific genes would miss the similarities of the responses between the two cell types. Therefore, while the number of DEGs is convenient for a cursory description of a certain cell type’s response, a determination of the pathways in which these DEGs participate is needed for a deeper understanding.

In many papers, DEGs are described as being caused by the “dysregulation” of gene expression, implying that the changes are the result of a “dysfunction” of gene regulation. However, if gene expression regulation was dysfunctional, it would not result in coordinated changes and genes would not pass statistical testing for differential expression (i.e., no DEGs). Therefore, affected neurons with gene expression “dysfunction” would likely show very few DEGs and the DEGs would be random, unlikely to participate in a common molecular pathway. Moreover, gene expression dysfunction would not lead to conserved changes between different studies or between humans and mouse models. Importantly, this review has described examples of similar gene expression responses between different studies (i.e., in the HD section) or different models within a study (i.e., the FFI and gCJD study). Therefore, these are programmed responses and not the breakdown of gene expression control. It is easy to envision that DEGs in resistant neurons were changed as a protective response. The same logic could be applied to vulnerable neurons, where the response was protective but not sufficient for full protection. In support of this conjecture, we described examples where vulnerable and resistant neurons had similar changes (the HD studies and the second ALS study [38]). This is important because an understanding of whether changes are protective or harmful is needed to apply the results of these studies to therapeutic development.

## 8. Future Directions

There has been a swift evolution in sc/snRNAseq techniques. A pioneering development in this domain is the Scissor algorithm (Single-Cell Identification of Subpopulations with Bulk Sample Phenotype Correlation) [102]. This tool was designed to merge and correlate bulk RNAseq data with scRNAseq information. The utility of Scissor is underscored by its capacity to harness phenotype data from bulk RNA, subsequently pinpointing relevant individual cells. This facilitates the nuanced identification of cell subgroups that might be pivotal drivers of diseases.

While technologies like CST and sc/snRNAseq offer deep insights, they come with limitations, notably the loss of spatial context due to tissue processing. Addressing this gap are innovative methods collectively termed spatial transcriptomics. These techniques have the capacity to detect a vast array of transcripts from tissue samples in a parallel manner. An intriguing adaptation of this approach specifically targets the translatome [103]. While these cutting-edge tools can identify up to 5000 genes, they demand significant computational power and advanced equipment—surpassing the requirements for CST or sc/snRNAseq [103]. The technical complexities of these methods might explain their limited application in ND studies so far.

Diving deeper into specialized methodologies, bacTRAP and RiboTag stand out for their unique capability to selectively capture the mRNAs associated with ribosomes, offering a real-time snapshot of protein synthesis. However, the spectrum of gene expression regulation is broad, and to fully grasp its nuances other layers of its control warrant exploration. A case in point is the study of miRNAs from MNs in SOD1^G93A^ mice [104], which was conducted using miRAP—a method reminiscent of RiboTag [105,106]. Unlike RiboTag, which uses an epitope-tagged ribosomal protein, miRAP employs an epitope-tagged Argonaute2, targeting the RNA-induced silencing complex. From this work, miRNA-218 emerged as a potential marker for resistant MNs, but how it connects to translatome alterations remains unknown. However, such an analysis, plus the study of other levels of gene expression, is possible with Tagger mice [113]. In Tagger mice, RiboTag is expressed in parallel with three additional proteins, including an epitope-tagged Argonaute2, a fluorescent protein targeted to the nucleus, and an enzyme that activates a uracil analog to timestamp newly synthesized RNA molecules [113].

In concluding this forward-looking review, it is evident that leveraging the power of bacTRAP, RiboTag, and Tagger, among other tools, will substantially expand our understanding of NDs. These tools are poised to unravel the intricate dynamics of selective vulnerability and resistance in neurons, charting a course toward the development of transformative therapeutic interventions.

## Figures and Tables

**Table 1:** Details of the studies in the focus of this review.

PMID	Authors	Disease	Method	Model or Source	Investigated Brain Region and Cell Types
26621731	Sun et al., 2015	ALS	bacTRAP	loxSOD1^G37R^	Spinal cord: motor neurons, astrocytes and oligodendrocytes
35320722	Moya et al., 2022	ALS	bacTRAP	SOD1^G93A^	Motor cortex, layer 5b: Colgalt2+ and Gprin3+ motor neurons
35960762	Kaczmarczyk et al., 2022	acquired prion	RiboTag	RML model of mouse-adapted scrapie, 10 and 18 weeks after infection	Hemibrain: astrocytes and Gad2+, vGluT2+, PV+ and SST+ neurons
36192034	Bauer et al., 2022	gCJD and FFI	RiboTag	KI-3F4-CJD and KI-3F4-FFI mice, age 9 months	cerebellum: Gad2+ and vGLuT2+ neurons
					cerebrum: Gad2+, vGluT2+, PV+ and SST+ neurons
26900923	Langfelder et al., 2016	HD	Bulk RNAseq	Allelic series HdhQ KI mice (Q20, Q80, Q92, Q111, Q140, Q175) at 2, 6, and 10 months	striatum, cortex
				Q175, 6 months	various brain and peripheral tissues
32681824	Lee et al., 2020	HD	bacTRAP	Allelic series HdhQ KI mice: Q20, Q50, Q111, Q170, 3 or 6 months	striatum: dSPNs, iSPNs, astroglia, cholinergic interneurons
			bacTRAP	zQ175DN KI-mice, 3 or 6 months	striatum: dSPNs, iSPNs, astroglia, cholinergic interneurons
			snRNAseq	zQ175DN KI-mice, 3 or 6 months	striatum
			snRNAseq	human postmortem, HD grade 2–4	caudate, putamen
34011527	Malaiya et al., 2021	HD	snRNAseq	zQ175DN KI-mice, aged 14–15 months	striatum
36670467	Bauer et al., 2023	HD	RiboTag	HdhQ200 KI-mice, aged 9 months	cerebellum: Gad2+ and vGLuT2+ neurons
					cerebrum: Gad2+, vGluT2+, PV+ neurons
32603655	Roussarie et al., 2020	AD	bacTRAP	wild-type mice, aged 5, 12, and 24 months	excitatory neurons in hippocampal CA1, CA2, CA3, and DG, and cortical ECII, S1 and V1.
				APP/PS1 Tg mice (Borchelt model) 6 months	excitatory neurons in ECII
31042697	Mathys et al., 2019	AD	snRNAseq	human postmortem, early and late stages of AB neuropathology	prefrontal cortex
33432193	Leng et al., 2021	AD	snRNAseq	human postmortem, early and late stages of tau neuropathology	entorhinal cortex and superior frontal gyrus

**Table 2:** Comparison and contrast of CST and scRNAseq/snRNAseq.

Feature	CST	scRNAseq/snRNAseq
Typical number of genes detected in ND studies	15,000 to 18,000	1000 to 8000
Cell populations	Mix of related cells	Unmixed individual cells
Transgene-dependent	Yes	No
Functional with frozen tissue	Yes	scRNAseq = no; snRNAseq = yes
RNA biotype studied	Translatome	mRNA transcriptome
